# Essentials of the Physical Diagnosis of Thoracic Diseases

**Published:** 1887-04

**Authors:** Frank Billings


ESSENTIALS OF THE PHYSICAL DIAGNOSIS OF THORACIC DISEASES. *By* E. DARWIN HUDSON, JR., A.M., M.D., *Professor of General Medicine and Diseases of the Chest in the New York Polyclinic, Physician to Bellevue and St. Elizabeth Hospitals. Pp. 63. New York:* STILES & CASH.

This manual was prepared for the use of Professor Hudson's students at the New York Polyclinic, and is of great assistance to them as a guide to his clinical lectures. It can be of but little value to others, because no one can do justice to so vast a subject within so small a space as the author devotes to it. It contains nothing but what is more satisfactorily explained in the larger text-books.

A careless error occurs on page six, where the apex of the right ventricle is placed in the right mammary region.

"Determining the situation and size of surrounding organs," is given as a *method* of physical diagnosis.

"Bronchial fremitus, produced by bronchitis;" "pitch refers to length of sound-wave;" "over gases pitch is very low, indeed;" "it takes four times as long for the blood to pass through the systematic as through the pulmonary circulation;" "the crepitant *râle* is heard only in two diseases, viz: croupous pneumonia and œdema of lungs," are statements partly or wholly erroneous.

FRANK BILLINGS.

